# Supplementary material for: Analysis of PPARγ Signaling Activity in Psoriasis
Source: Int J Mol Sci. 2021 Aug 10;22(16):8603. doi: 10.3390/ijms22168603 (PMC8395241; doi:10.3390/ijms22168603)
Supplement: Supplementary file 1 [file ijms-22-08603-s001.zip › Supplemental materials_Analysis of PPARg signaling activity in psoriasis/Pathway models/Models images and html files/PPARG inhibits phenotypes assotiated with psoriasis/237471.html]

alcohol drinking


# Disease alcohol drinking

|  |  |
| --- | --- |
| URN | urn:agi-meshdis:Alcohol%20Drinking |
| Total Entities | 3 |
| Connectivity | 4741 |
| Name | alcohol drinking |

---

|  |  |
| --- | --- |
| ChildConcepts | binge drinking |
|  | underage drinking |
|  | college drinking |

---

|  |  |
| --- | --- |
| Pathway | PPARG inhibits phenotypes assotiated with psoriasis |

---

|  |  |
| --- | --- |
| MedScan ID | 9005528 |

---

|  |  |
| --- | --- |
| Alias | EtOH consumption |
|  | Alcohol Consumption |
|  | EtOH intakes |
|  | ethanol intake |
|  | ethanol consumption |
|  | ethanol (EtOH) intakes |
|  | Alcohol Use |
|  | intake of alcohol |
|  | ethanol (EtOH) consumption |
|  | Consumptions, Alcohol |
|  | intakes of ethanol |
|  | alcohol ingestion |
|  | uses of alcohol |
|  | Drinking, Alcohol |
|  | Alcohol Uses |
|  | intakes of alcohol |
|  | alcohol ingestions |
|  | alcohol intakes |
|  | ethanol (EtOH) consumptions |
|  | ethanol (EtOH) drinking |
|  | ethanol intakes |
|  | alcohol consumptions |
|  | EtOH intake |
|  | ethanol (EtOH) intake |
|  | Alcohol Drinking |
|  | ethanol consumptions |
|  | use of alcohol |
|  | consumption of ethanol |
|  | intake of ethanol |
|  | alcohol intake |
|  | EtOH consumptions |
|  | Consumption, Alcohol |
|  | EtOH drinking |

---

|  |  |
| --- | --- |
| MeSH ID | D000428 |
|  | M0000650 |

---

|  |  |
| --- | --- |
| MeSH Heading | Alcohol Drinking |

---
